# A case report of pulmonary adenocarcinoma with thoracic duct lymphangioma and chylothorax

**DOI:** 10.3389/fmed.2025.1712061

**Published:** 2025-11-03

**Authors:** Zichun Tang, Rui Xu, Peng Dai, Juncheng Chen, Zongwei Xiao

**Affiliations:** ^1^Department of Cardio-Thoracic Surgery, North Sichuan Medical College, Nanchong, Sichuan, China; ^2^Department of Neurosurgery, North Sichuan Medical College, Nanchong, Sichuan, China; ^3^Department of Cardio-Thoracic Surgery, Chengdu Second People’s Hospital, Chengdu, Sichuan, China

**Keywords:** lung adenocarcinoma, mediastinal tumor, ductus thoracicus, lymphangioma, chylothorax

## Abstract

Thoracic duct lymphangioma, a rare benign lesion, requires surgical resection. The thoracic duct is highly susceptible to damage during surgery, leading to chylothorax. Therefore, meticulous exploration and gradual dissection are crucial to minimizing this risk. Thoracic duct lymphangioma coexisting with lung adenocarcinoma is clinically rare. Preoperative imaging evaluation should be comprehensive, intraoperative meticulous dissection is crucial to reduce the likelihood of thoracic duct injury, and early intervention for postoperative chylothorax is essential to improve prognosis. This case aims to investigate the clinical characteristics, diagnostic and therapeutic strategies, and prognosis of lung adenocarcinoma complicated with thoracic duct lymphangioma and chylothorax. This case report aims to provide clinical reference for managing such complex cases.

## Introduction

Mediastinal cystic lymphangiomas account for only 0.7%–4% of all mediastinal tumor and are usually asymptomatic (1). Thoracic-duct lymphangioma is vanishingly rare; its imaging signature is so subtle that it is repeatedly misdiagnosed as a foregut duplication cyst, necrotic lymph-node metastasis, or even a simple pleural effusion. As the thoracic duct transports most of the body’s lymphatic fluid, even trivial injury can precipitate high output chylothorax within hours, converting an elective resection into an emergency (2).

Non-small-cell lung cancer (NSCLC) is the commonest thoracic malignancy, and lung adenocarcinoma comprises 40%–50% of these cases (3). The simultaneous presence of thoracic duct lymphangioma and lung adenocarcinoma has been reported only sporadically, so no standardized surgical protocol exists. The oncologic imperative for complete lymph-node dissection directly conflicts with the need to preserve a malformed duct that may be the sole drainage pathway for the entire abdominal lymphatic system. Consequently, any resection strategy must integrate precise pre-operative mapping of the duct, immediate intra-operative recognition of its aberrant course, and a low threshold for prophylactic ligation or micro-vascular reconstruction.

Chylous ascites, although more often ascribed to cirrhosis, trauma, or lymphoma, can also arise from downstream obstruction of the thoracic duct by an adjacent malignancy or by the lymphangioma itself. When the two pathologies coexist, the risk of postoperative chylothorax is compounded by the possibility of simultaneous chylous ascites, demanding a multidisciplinary plan that spans pre-operative nutritional optimization, intra-operative lymphatic imaging, and prompt escalation to somatostatin analogs or surgical re-intervention at the first sign of persistent leak (4, 5). We describe a patient in whom an incidentally discovered mediastinal lymphangioma was found contiguous with a right-lower-lobe adenocarcinoma, outline the diagnostic work-up that clarified the dual pathology, and detail the integrated strategy that achieved oncologic clearance while averting catastrophic chyle leak.

## Case report

**FIGURE 1:**
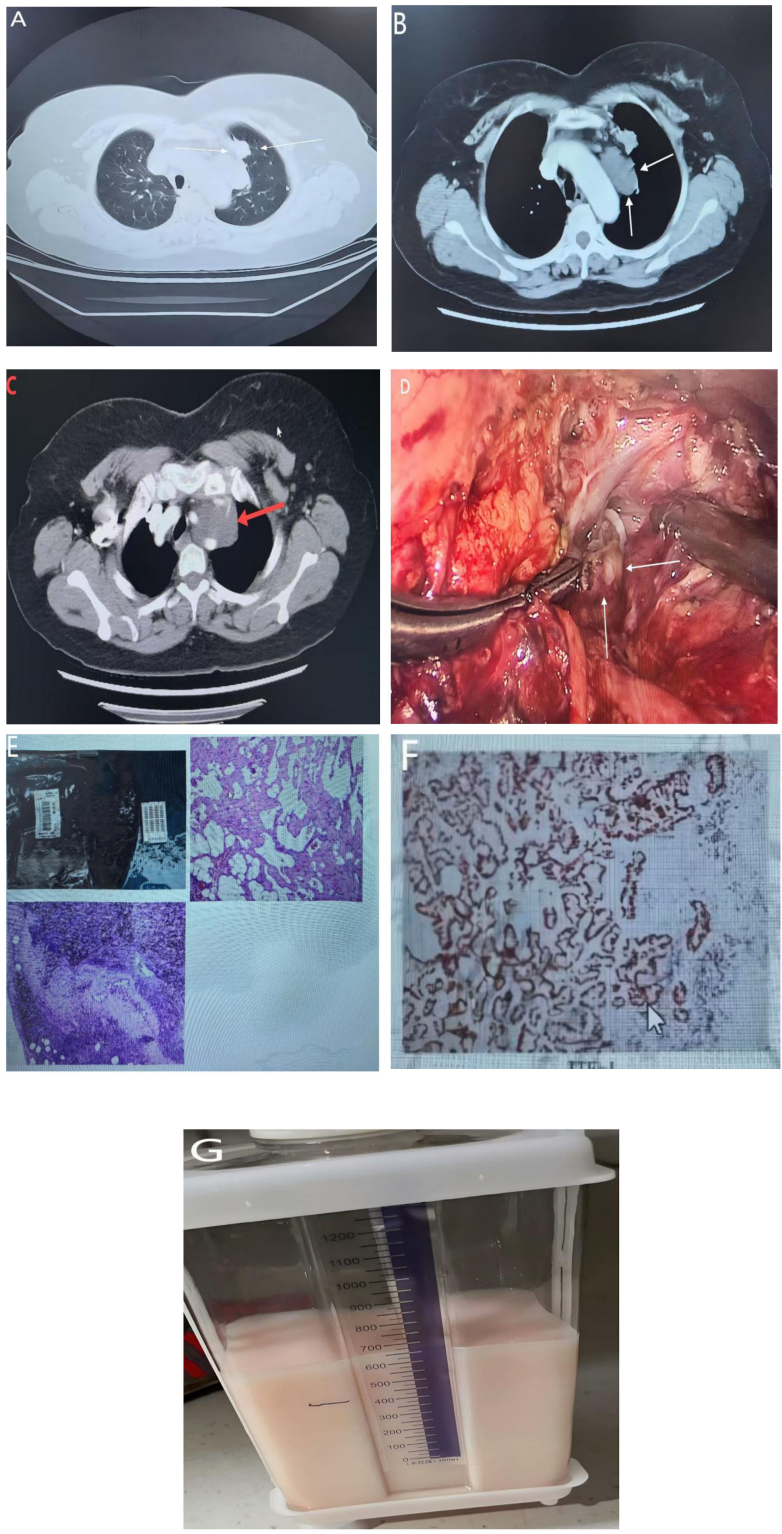
**(A)** A contrast-enhanced chest CT demonstrating a solid nodule abutting the mediastinal pleura in the anterior segment of the left upper lobe, highly suspicious for malignancy. **(B)** Axial and **(C)** coronal reveal a well-circumscribed, low-density, cystic mass in the superior mediastinum consistent with a lymphatic malformation. **(D)** Depicts the intra-operative view of thoracic-duct fistula ligation performed via right thoracotomy. **(E)** Displays the gross surgical specimen of the left upper-lobe pulmonary nodule. **(F)** The resected mediastinal cystic mass; histopathology confirmed a vascular tumor compatible with lymphangioma. **(G)** Illustrates the immediate postoperative chest-tube drainage of large-volume chylous fluid.

A 60-year-old female patient was admitted to the Department of Thoracic and Cardiovascular Surgery at Chengdu Second People’s Hospital on December 17, 2024, with a chief complaint of “recurrent cough for over 1 year and incidental discovery of a left upper lobe lung nodule for 1 week.” Physical examination upon admission revealed no abnormal signs. She had a 2-year history of hypertension, managed with regular medication but with suboptimal control. She had no history of smoking. Contrast-enhanced chest CT on 17 December 2024 showed a solid juxta mediastinal nodule in the anterior segment of the left upper lobe highly suggestive of malignancy, accompanied by multiple mildly enlarged mediastinal lymph nodes and an indeterminate cystic-density mediastinal mass; scattered bilateral pulmonary micro-nodules (largest ground-glass opacity at the right upper-lobe apex), bronchitic changes, chronic inflammatory foci, cardiomegaly, and mild bilateral pleural thickening were also noted, with no other abnormalities (Figures 1A–D).

### Treatment course & pathology

Four days after admission, on December 19, 2024, the patient underwent a video-assisted thoracoscopic (VATS) left upper lobectomy with radical lung cancer resection, mediastinal mass excision, and thoracic duct ligation. Three ports were placed: an observation port at the 7th intercostal space along the left mid-axillary line and working ports at the 4th intercostal space anterior axillary line and 9th intercostal space posterior axillary line. Intraoperatively, a left upper lobe mass with pleural puckering was identified. A wedge resection was performed, and frozen section confirmed moderately differentiated invasive adenocarcinoma. The procedure proceeded with completion of the lobectomy and systematic lymph node dissection (stations 5, 6, 9, 10). A large cystic-solid mediastinal mass was encountered anteriorly, adherent to the phrenic and vagus nerves and left innominate vein. Meticulous dissection preserved these structures. Upon cyst incision, copious chylous-like fluid was noted, and a ruptured, tortuously dilated thoracic duct was identified, suggesting a congenital thoracic duct malformation. Attempted suture ligation of the duct reduced but did not eliminate leakage. Postoperatively, persistent high output chylothorax (Figure 1G) necessitated a second VATS procedure on January 15, 2025, for thoracic duct fistula closure, after which recovery was uneventful.

Pathology (Figures 1E, F) revealed:

#### Mediastinal mass

Lymphatic tumor consistent with lymphangioma, with negative lymph node metastases. Immunohistochemistry (LIHC2401239): D2-40(+), CD34(+), HMB45(−), Desmin (+), ER (−), Ki-67 (low).

#### Left upper lobe nodule

Moderately differentiated invasive adenocarcinoma (2 cm × 2 cm × 1.2 cm), invading visceral pleura (PL2) with focal penetration, multifocal vascular invasion, and lymphatic tumor emboli. Immunohistochemistry (LIHC2401226): TTF-1(+), Napsin A(+), CK7(+), CDX-2(−), CK20(−), CK5/6(−), P40(−), Ki-67 (∼20%); CD34 and D2-40 confirmed vascular invasion. Special stains (LR2400307): Elastic fiber staining confirmed pleural invasion; AB staining showed intracellular mucin in rare tumor cells.

## Discussion

Lymphatic channels that originate within the peritoneal cavity converge posterior to the aorta, just below the diaphragm, to form the thoracic duct. This conduit measures 36–45 cm in length and 2–3 mm in diameter. Ascending alongside the aorta, it penetrates the diaphragmatic hiatus, then ascends through the posterior mediastinum on the right of the vertebral column. At the T3–T4 level it arches leftward, crosses the midline, and ascends behind the esophagus into the superior mediastinum. In the neck it curves laterally to terminate at the left venous angle, emptying into the subclavian vein. This “classical” anatomy is present in only 65% of individuals; embryologic variants—duplications, plexiform networks, or aberrant terminations—account for the duct’s vulnerability to iatrogenic injury despite meticulous surgical technique. Numerous bicuspid valves ensure unidirectional chyle flow, while the respiratory pump augments propulsion toward the systemic circulation (2).

Thoracic duct lymphangioma is an exceedingly rare mediastinal tumor whose subtle clinical picture usually leads to incidental discovery on imaging (5). In the present case, contrast-enhanced chest CT showed a mediastinal cystic mass that could not be characterized pre-operatively; definitive histology after resection identified lymphangioma, matching previous descriptions of well-circumscribed cystic lesions that are easily mistaken for other mediastinal cysts (5). Lung adenocarcinoma—the commonest NSCLC subtype (3)—and thoracic-duct lymphangioma have poorly understood pathogeneses. Although congenital lymphatic malformation or secondary lymphatic injury has been proposed (6), no direct evidence links lung adenocarcinoma to lymphangioma formation. Nevertheless, tumor-derived lymphangiogenic factors such as VEGF-C could stimulate local lymphatic proliferation (7), an association that merits further study. Thoracic-duct injury is a serious surgical hazard; resultant chylothorax, though uncommon, can precipitate malnutrition and immunosuppression if not promptly managed (8). Recent advancements include preoperative lymphangiography combined with intraoperative indocyanine green (ICG) fluorescence imaging for thoracic duct localization, significantly reducing intraoperative injury risk. Postoperative chylothorax management depends on drainage volume: conservative therapy (medium-chain triglyceride diet, octreotide) suffices for low output, while persistent chylothorax (>1000 mL/day) mandates early surgical intervention (9). Multidisciplinary collaboration (thoracic surgery, radiology, pathology, nutrition) is critical for such complex cases. Preoperative precision assessment (dynamic contrast-enhanced MRI or CT lymphangiography) aids individualized surgical planning (10), while postoperative nutritional support (high-protein, low-fat diet with parenteral nutrition) is vital for recovery. This patient achieved favorable outcomes after tumor resection and chylothorax management but requires long-term surveillance for tumor recurrence and lymphangioma residuals. When coexisting with lung adenocarcinoma, priority is given to malignancy treatment, though mediastinal masses may heighten thoracic duct injury risk. The high postoperative chylous output in this case underscores the need for thorough intraoperative thoracic duct exploration. Key lessons include routine preoperative chest CT and echocardiography, with chest MRI if indicated. Studies show complete lymphangioma resection yields low recurrence rates, while lung adenocarcinoma prognosis hinges on pathological stage and molecular features (11). For this patient (pT2aN0M0, lymph vascular invasion positive), adjuvant therapy (targeted therapy or chemotherapy) may further improve outcomes (12).

## Conclusion

The management of concomitant thoracic duct lymphangioma and lung adenocarcinoma requires a dual focus on oncological radicality and meticulous anatomical preservation. Intraoperative precision and early postoperative intervention for chylothorax are critical to optimizing outcomes.

## Data Availability

The original contributions presented in this study are included in this article/supplementary material, further inquiries can be directed to the corresponding author.
